# Over-Expression of HSP47 Augments Mouse Embryonic Stem Cell Smooth Muscle Differentiation and Chemotaxis

**DOI:** 10.1371/journal.pone.0086118

**Published:** 2014-01-16

**Authors:** Mei Mei Wong, Xiaoke Yin, Claire Potter, Baoqi Yu, Hao Cai, Elisabetta Di Bernardini, Qingbo Xu

**Affiliations:** Cardiovascular Division, King's College London, British Heart Foundation Centre, London, United Kingdom; William Harvey Research Institute, Barts and The London School of Medicine and Dentistry, Queen Mary University of London, United Kingdom

## Abstract

In the recent decade, embryonic stem cells (ESC) have emerged as an attractive cell source of smooth muscle cells (SMC) for vascular tissue engineering owing to their unlimited self-renewal and differentiation capacities. Despite their promise in therapy, their efficacy is still hampered by the lack of definitive SMC differentiation mechanisms and difficulties in successful trafficking of the ESC towards a site of injury or target tissue. Heat shock protein 47 (HSP47) is a 47-kDa molecular chaperone that is required for the maturation of various types of collagen and has been shown to be a critical modulator of different pathological and physiological processes. To date, the role of HSP47 on ESC to SMC differentiation or ESC chemotaxis is not known and may represent a potential molecular approach by which ESC can be manipulated to increase their efficacy in clinic. We provide evidence that HSP47 is highly expressed during ESC differentiation into the SMC lineage and that HSP47 reduction results in an attenuation of the differentiation. Our experiments using a HSP47 plasmid transfection system show that gene over-expression is sufficient to induce ESC-SMC differentiation, even in the absence of exogenous stimuli. Furthermore, HSP47 over-expression in ESC also increases their chemotaxis and migratory responses towards a panel of chemokines, likely via the upregulation of chemokine receptors. Our findings provide direct evidence of induced ESC migration and differentiation into SMC via the over-expression of HSP47, thus identifying a novel approach of molecular manipulation that can potentially be exploited to improve stem cell therapy for vascular repair and regeneration.

## Introduction

The construction of vascular tissues to replace damaged and injured vessels is a fundamental strategy in development of new treatments for cardiovascular disease. The use of mature endothelial and smooth muscle cells (SMC) is ideal for the repair of diseased vessels. SMC, in particular, are the most abundant cell type within blood vessels and are known to play a key role in maintenance of the vasculature [1]. Nevertheless, the exploitation of these vascular cells for therapy is often limited, due to insufficient supply of the cells, as they can only divide for a finite number of times before undergoing growth arrest and senescence.

In recent years, stem cells have emerged as an attractive cell source for vascular tissue engineering owing to their unlimited self-renewal and differentiation capacities. Indeed cell types such as embryonic stem cells (ESC), induced (or partially induced) pluripotent stem cells (iPS or PiPs) have been documented for their capacity to differentiate into functional SMC [2], [3], [4]. Furthermore, several novel mechanisms have been postulated to be involved in driving the differentiation process i.e. extracellular signal-regulated kinase (ERK)/β-catenin [5], dickkopf-related protein-3 (DKK-3) [2], reactive oxygen species [6], histone deacetylases 7 [7], microRNA (miRNA)-221 and miRNA-25 [3]. Despite the existing data, a clear consensus has not been reached and an abundance of studies are still ongoing with the aim to improve the efficacy of stem cells in translational and clinical studies.

Although stem cells are promising tools in the cardiovascular regeneration field, their efficacy is very often hampered by the lack of successful trafficking of the cells towards a site of injury or target tissue [8]. Furthermore, effective transdifferentiation of stem cells is likely to be highly dependent on their regulated chemotaxis, whether via the administration of exogenous factors or molecular programming of the stem cells. Chemokine receptors and their corresponding ligands have been established as key mediators of migration and trafficking in many cell types [9], [10]. For instance, the CXCR4/stromal-derived factor-1 (SDF-1) axis has been documented to play a critical role in initiating stem cell movement, in particular that of adventitial stem cells [11], endothelial progenitor cells (EPC) and haematopoietic stem cells (HSC) [12]. To date the chemokine receptor expression profile, of the aforementioned stem cell types, with respect to cardiovascular regeneration remains poorly defined. Identification of the precise means by which chemokine receptor expression can be manipulated in stem cells is likely to improve their delivery towards target tissues.

Heat shock proteins (HSP) are a class of evolutionarily conserved proteins that can be expressed under the influence of various types of stress, thus allowing them to be critical modulators of different pathological and physiological processes [13]. HSP47, or SERPINH1, is a 47-kDa HSP that acts as a molecular chaperone required for proper assembly of triple-helical pro-collagen molecules, which are eventually transported into extracellular space via the Golgi apparatus [14]. While previous studies show increased expression of HSP47 under several pathological conditions, such as idiopathic pulmonary fibrosis [15], renal scarring [16], neointima formation [17] and glomerulosclerosis [18], the protein has also been found to act as therapeutic mediators that suppress cytotoxicity by reducing abnormally aggregated or misfolded procollagen molecules [19]. Presently, the molecular and functional roles of HSP47 in stem cell differentiation and migration remain unknown.

In this study, we provide novel evidence that endoplasmic reticulum (ER)-resident HSP47 is highly expressed during mouse ESC differentiation into SMC and that lack of HSP47 results in a complete attenuation of differentiation. Furthermore, the over-expression of HSP47 is sufficient to drive ESC-SMC differentiation in the absence of any exogenous stimulation. More intriguingly, our study provides the first evidence that the over-expression of HSP47 also increases ESC chemotaxis and their responses towards chemokines, likely via the upregulation of chemokine receptors. Together, our study suggests a putative role of HSP47 in enhancing the chemotaxis and differentiation of ESC into SMC, thus indicating a novel mechanism of molecular manipulation that can be exploited to improve stem cell therapy for vascular repair and regeneration.

## Experimental Procedures

### Materials

Cell culture media, serum and cell culture supplements were purchased from ATCC, Millipore, Gibco and PAA. Antibodies against Calponin, Smooth Muscle 22-α (SM-22α) and Smooth Muscle-Myosin Heavy Chain II (SM-MyHCII) were purchased from Abcam. Antibodies against Smooth Muscle-α Actin (SM-αA) and Glyceraldehyde 3-phosphate dehydrogenase (GAPDH) was purchased from Sigma Aldrich. Antibodies against HSP47 were purchased from MBL International and Millipore. Secondary antibodies for immunostaining anti-mouse Alexa488, anti-rabbit Alexa488 and anti-rabbit Alexa594 were purchased from Invitrogen, whereas secondary antibodies for Western Blotting were purchased from Dako.

### Embryonic Stem Cell Culture and Differentiation

Mouse ESC were purchased as a cell line (ES-D3, ATCC) and cultured in gelatin-coated flasks (Sigma-Aldrich) in DMEM (ATCC) medium with 10% FBS, leukemia inhibitory factor (10 ng/ml) and 0.1 mM 2-mercaptoethanol. The ESC were differentiated into SMC by culturing on Collagen IV for different lengths of time as stated in α-MEM (Gibco) with 10% FBS and 0.5 mM 2-mercapotoethanol.

### Quantitative RT-PCR

Total RNA was isolated from ESC using an RNeasy Mini kit (QIAGEN Inc.) according to manufacturer's instructions. In brief, 2 µg RNA were reverse transcribed into cDNA with random primers by MMLV reverse transcriptase (RT) (Promega) and real time RT-PCR was performed using 2 ng of cDNA per sample with a SYBR Green Master Mix (Promega) in a 25-μl reaction. For each sample, Ct values were measured using ABI PRISM 7000 Sequence Detector (Applied Biosystems) and 18 S ribosomal RNA was used as an endogenous control for normalizing the amounts of RNA. Sequences of mouse SMC and chemokine receptor primer sets used were as previously described by our laboratory [5], [20]. Sequences of the mouse HSP47 primer set are as follows. HSP47: forward5′>GCAGCAGCAAGCAACACTACAACT<3′,reverse5′>AGAACATGGCGTTCACAAGCAGT G<3′.

### Western Blot Analysis

Embryonic stem cells were harvested and lysed with IP-A buffer (25 mM Tris-HCl pH 7.5, 150 mM NaCl, 1 mM EDTA pH 8.0, 1%Triton X-100 plus protease inhibitors) and proteins were sequentially measured using the Bradford method. ESC lysates (40 µg) were applied to an acrylamide gel using SDS-PAGE and transferred to a nitrocellulose membrane (Amersham Biosciences), followed by a standard western blotting procedure. Polyclonal antibodies against SM-22α, calponin (both purchased from Abcam, UK) GAPDH, SM-αA (both from Sigma Alrich) and HSP47 (MBL, International Corporation, Woburn MA) were used to detect the respective proteins. A HRP-conjugated secondary antibody and an ECL detection system (Amersham Biosciences) were used to detect bound primary antibodies.

### Immunofluorescence Staining

Cultures of ESC were fixed with 4% paraformaldehyde, permeabilized with 0.1% Triton X-100 in PBS and blocked with 10% normal swine serum (Dako). Incubation of cell or tissue samples with primary antibodies was performed at 4°C overnight, followed by incubation with secondary antibodies for 30 mins at 37°C after three thorough washes with PBS. Subsequently, the cells were counterstained with DAPI (1:1000 in PBS) for 3 mins at room temperature and mounted with fluorescent mounting media (Dako) before image acquisition using an Axio Imager. M2 microscope and AxioVision Digital Imaging System (Carl Zeiss Ltd.). Primary antibodies used were Calponin, SM-22α (both from Abcam), SM-αA (Sigma Aldrich), HSP47 (Abcam) and DAPI (Sigma Aldrich). The appropriate fluorescent-conjugated (Alexa 488 and Alexa 546) IgG antibodies were used as secondary antibodies (Invitrogen).

### Transwell Chemotaxis Assay

Chemotaxis assays were performed with transwell inserts of 5.0 micron membrane pore size (Becton Dickinson Labware, USA). ESC were harvested using trypsin-EDTA and subsequently loaded onto the upper chamber at 1×10^5^ cells in serum free media, whereas the bottom chamber contained serum free media with either FBS (20%), eotaxin, KC, MCP-1, Rantes or SDF-1 (all chemokines at 10 ng/ml). After an overnight incubation, non-migrating ESC on the upper side of the filters were removed using a cotton tip applicator, whereas ESC on the underside of the membrane were fixed with 3% PFA for 10 mins before staining with 1% crystal violet solution (diluted with dH_2_0) at room temperature for 15 mins. Data was expressed as the mean number of migrated ESC in 5 random fields of view (at 20×). Chemotaxis assays were also carried out on cells following HSP47 over-expression.

### HSP47 Over-expression

For transient over-expression of HSP47, 1.0 µg per 1×10^6^ ESC of pCMV6-HSP47 (Origene) expression plasmid was introduced into ESC by nucleofector II (Amaxa, Germany) with a mouse ESC nucleofection kit (Amaxa, VPH-1001). The program A-23 was used according to manufacturer's instructions. An empty vector (Addgene) plasmid was included as a negative control (mock). Total ESC RNA and proteins were harvested after gene over-expression and subjected to real time RT-PCR or western blot analysis, respectively.

### HSP47 Knockdown

Gene suppression of HSP47 (NM_009825.1) was carried out using MISSION short hairpin RNA (shRNA) lentiviral plasmids transfer as previously described [21]. The non-targeting vector, SHC002 was used as a negative control. All shRNA lentiviral plasmids were purchased from Sigma Aldrich UK. Total RNA or proteins were harvested after gene ablation and subjected to real time RT-PCR or western blot analysis, respectively.

### Statistical Analysis

Data in this study represent mean and standard error of the mean (S.E.M.) of at least three separate experiments. Statistical analysis was performed using Graphpad Prism V.4 (GraphPad Software, San Diego CA) with analysis of variance (ANOVA) followed by Dunnett's multiple comparison tests. Significance was considered when p<0.05.

## Results

### HSP47 is expressed during SMC differentiation

**Figure 1:**
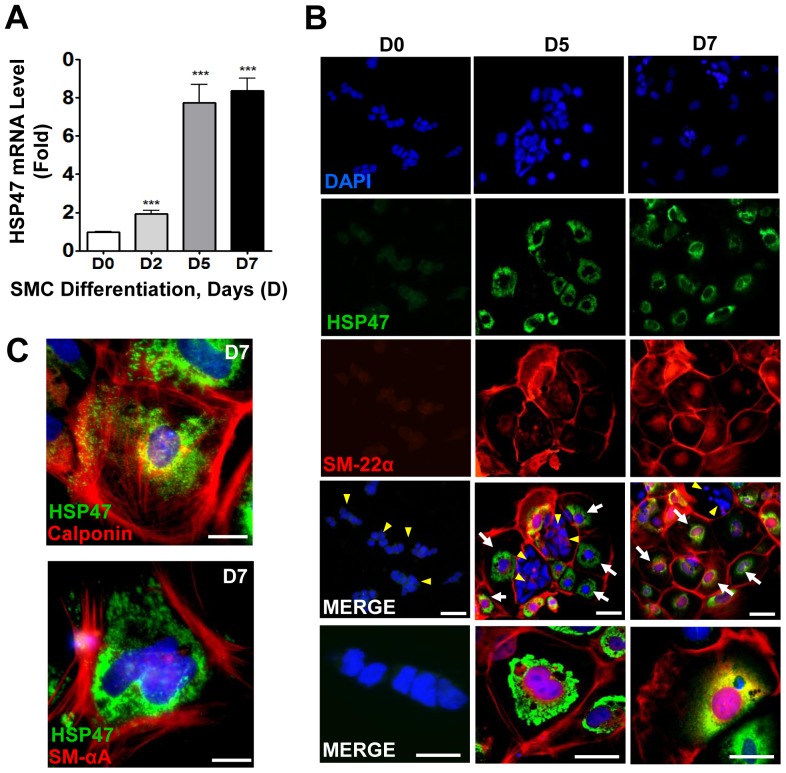
HSP47 is expressed during SMC differentiation of ESC. A–C, HSP47 is increased during ESC-SMC differentiation. Murine ESC were differentiated into SMC in the presence of Collagen IV for the time indicated, followed by quantitative RT-PCR analysis of HSP47 mRNA (A), immunofluorescent staining with HSP47 (green) and SM-22α (red) antibodies (B), calponin (red) and SM-αA (red) (C). DAPI was included to counterstain the nucleus (blue). Scale bar, 30 µm. Images shown are representative of at least three separate experiments, whereas graphs are shown as mean ± SEM of at least three independent experiments, ***P<0.005 compared with Day 0.

Firstly, we wanted to investigate if HSP47 was expressed during the process of SMC differentiation from ESC. Using a previously established differentiation protocol [22], mouse ESC were differentiated into functional SMC in the presence of Collagen IV. Over the course of differentiation, we found a time-dependent increase of the HSP47 gene that peaked at day 7 (Figure 1A). The upregulation of HSP47 was also confirmed at protein level by immunofluorescence staining with HSP47 (green) and SM-22α (red) antibodies (Figure 1B). Experiments also showed that expression of HSP47 increases in a time dependent manner and that it is localized specifically in the endoplasmic reticulum of the ESC (Figure 1). Intriguingly, HSP47 was only found on cells that also expressed the SMC marker (SM-22α) (indicated by white arrows), but not within clusters of undifferentiated ESC (indicated by yellow arrow heads). Consistently, HSP47 was also expressed concomitantly with other SMC markers such as calponin and SM-αA following 7 days of differentiation (Figure 1C). Thus, these data suggest that HSP47 is likely to play a role in mediating the differentiation of ESC into functional SMCs.

### Over-expression of HSP47 can induce stem cell-SMC differentiation

**Figure 2:**
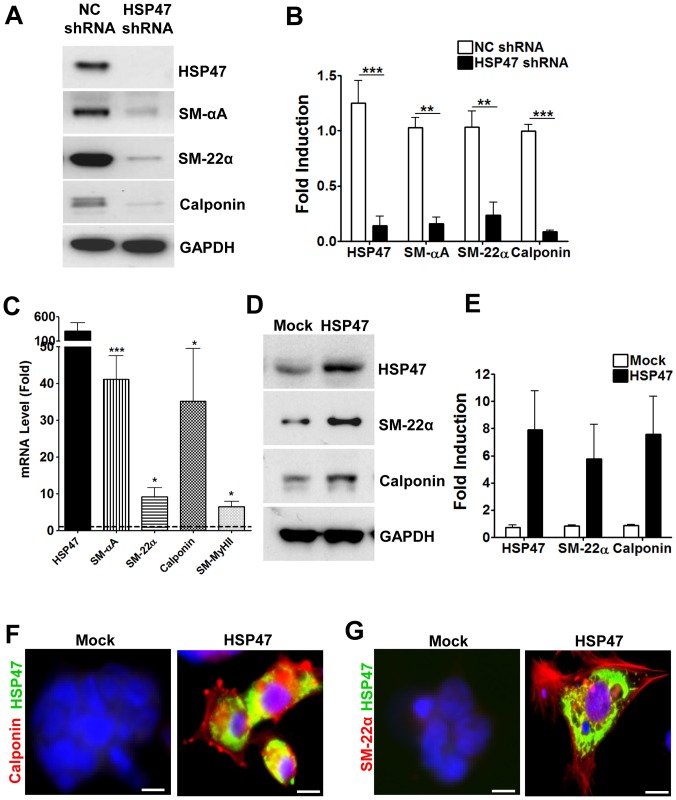
HSP47 can regulate ESC-SMC differentiation. A and B, knockdown of HSP47 abolishes HSP47 expression and attenuates ESC-SMC differentiation. ESCs were infected with non-coding (NC) or HSP47 shRNA lentiviruses for 24 h prior to SMC differentiation, followed by Western blot analysis (A) and quantification (B). C–E, over-expression of HSP47 induces SMC differentiation of ESC. ESCs were transfected either with a pCMV6-HSP47 or mock plasmid (1 µg/1×10^6^ cells) via electroporation, followed by real time RT-PCR (C), Western blotting (D, E) and Immunofluorescent staining with HSP47 (green), calponin (red) (F) and SM-22α (red) (G) antibodies. DAPI was included to counterstain the nucleus (blue). Scale bar, 30 µm. Images and blots shown are representative of at least three separate experiments, whereas graphs are shown as mean ± SEM of at least three independent experiments, *P<0.05, **P<0.01, ***P<0.005 fold increase compared with mock control, dotted line.

To confirm the role of HSP47 in mediating ESC-SMC differentiation, the gene was ablated in ESC using lentiviral shRNA knockdown prior to differentiation. Figure 2A and 2B show that knockdown of HSP47 results in a marked and significant ablation of SMC markers in response to Collagen IV-induced differentiation.

While experiments thus far involved SMC differentiation in response to an exogenous stimulus, we wondered whether over-expression of HSP47 itself is sufficient to induce ESC-SMC differentiation. For induction of transient HSP47 over-expression, a HSP47 expression plasmid was introduced into the ESC via nucleofection and maintained in normal culture conditions (without Collagen IV). ESC were also transfected with an empty vector plasmid (mock) as a control. Results show that HSP47-overexpressing ESC had significantly higher gene expression levels of a panel of SMC markers including SM-αA, SM-22α, calponin and SM-MyHCII (Figure 2C). The upregulation of SMC marker expression in HSP47-overexpressing cells was also evident at the protein level, as shown with western blotting (Figures 2D and 2E) and immunofluorescence staining (Figures 2F and 2G). These results show that the over-expression of HSP47 is sufficient to drive SMC differentiation of ESC, albeit the absence of exogenous stimulants such as Collagen IV.

### Over-expression of HSP47 induces stem cell chemotaxis

**Figure 3:**
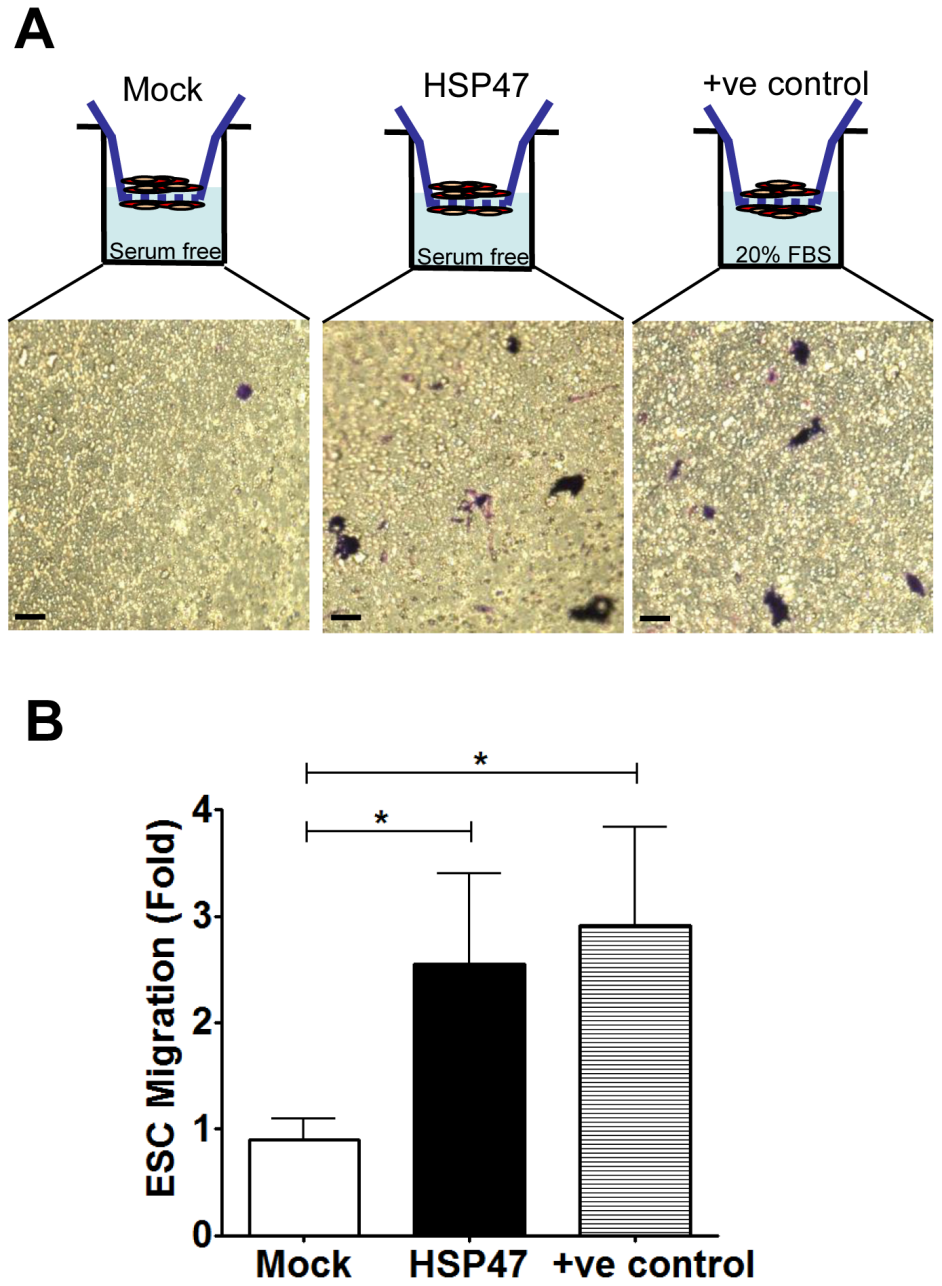
HSP47 over-expression induces ESC chemotaxis. A and B, over-expression of HSP47 induces chemotaxis of ESC. ESCs were transfected with pCMV6-HSP47 plasmid (1 µg/1×10^6^ cells) via electroporation and subjected to 5.0micron transwell chemotaxis assays 48 hours later. Chemotaxis of ESCs (either HSP47 or mock) towards serum free media in the transwells was documented following 1% crystal violet staining. (Scale bars, 30 µm) Chemotaxis of ESCs towards media containing 20% FBS was used as a positive control. Chemotaxis index was defined as the mean number of ESCs counted per 10 random fields of view with 20× objective and presented as fold increase compared to the mock control. Graphs are shown as mean ± SEM of three independent experiments. *p<0.05 compared with mock control.

The efficacy of ESC in therapy largely depends on their capacity of homing and localizing within tissues of interest. for repair and regeneration [8]. This concern prompted us to investigate whether, in light of its promising role in inducing the differentiation of ESC into potentially functional SMC, HSP47 could also play a role in inducing ESC migratory capacity. Therefore, we compared the chemotaxis of HSP47-stem cells to mock-stem cells using a 5.0 micron transwell system. Our data confirmed that HSP47-overexpressing ESC had markedly increased chemotaxis capacities compared to mock controls (Figure 3A). Furthermore, the increment that was seen was comparable to stem cell chemotaxis towards 20% FBS (established positive control) (Figure 3A). Quantitative analysis of these transwell assays revealed a significant increase in ESC chemotaxis following HSP47 over-expression (Figure 3B).

### HSP47 over-expression induces chemokine-mediated stem cell chemotaxis via induction of chemokine receptor expression

**Figure 4:**
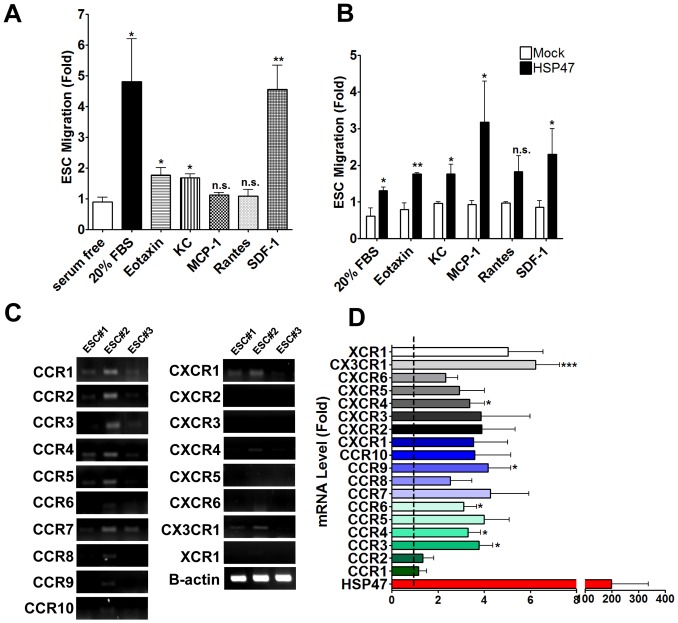
HSP47 over-expression enhances chemokine-mediated ESC chemotaxis and chemokine receptor expression. ESCs migrate in variable affinities in response to a restricted set of chemokines. Chemotaxis of ESCs towards serum free media containing either 20% FBS, eotaxin, KC, MCP-1, Rantes and SDF-1 (all chemokines at 10 ng/ml) in the transwells was documented following 1% crystal violet staining (A). Over-expression of HSP47 induces chemokine-mediated chemotaxis of ESC. Chemotaxis of ESCs (either HSP47 or mock) towards serum free media containing either 20% FBS, eotaxin, KC, MCP-1, Rantes and SDF-1 (all chemokines at 10 ng/ml) was documented following 1% crystal violet staining and represented graphically as a fold increase over mock ESC controls (B). *P<0.05, **P<0.01 compared with mock control. ESCs express a restricted set of chemokine receptor. ESCs cultured under normal culture conditions were harvested and subjected to routine RT-PCR for analysis of chemokine receptor expression (C). Over-expression of HSP47 induces ESC chemokine receptor expression. ESCs were transfected either with a pCMV6-HSP47 or mock plasmid (1 µg/1×10^6^ cells) via electroporation, followed by real time RT-PCR (D). Graphs are shown as mean ± SEM of at least three independent experiments, *P<0.05, ***P<0.005 fold increase compared with mock control, dotted line.

Chemokines and their corresponding receptors play critical roles in regulating the mobilization of many cell types [9], [10], [23], depending on the appropriate patho/physiological conditions that are present. Therefore, we wondered if the HSP47-induced migratory capacity of ESC could also enhance their tropism towards various chemokines. Notably, we found that in the absence of any stimulus or genetic manipulation, ESC were able to respond to a panel of chemokines at variable affinities; most of which were relatively low when compared to the positive control i.e. 20% FBS (Figure 4A). Intriguingly, we found that over-expression of HSP47 resulted in a super-induction of their migratory responses towards all the chemokines (eotaxin, keratinocyte chemokine (KC), monocyte chemotactic protein-1 (MCP-1), Rantes and SDF-1) and 20% FBS (Figure 4B). Interestingly, the enhancement of HSP47-stem cell chemotaxis was most apparent in response to MCP-1 and SDF-1, both of which have been shown to induce vascular stem/progenitor cell recruitment [11], [24].

**Figure 5:**
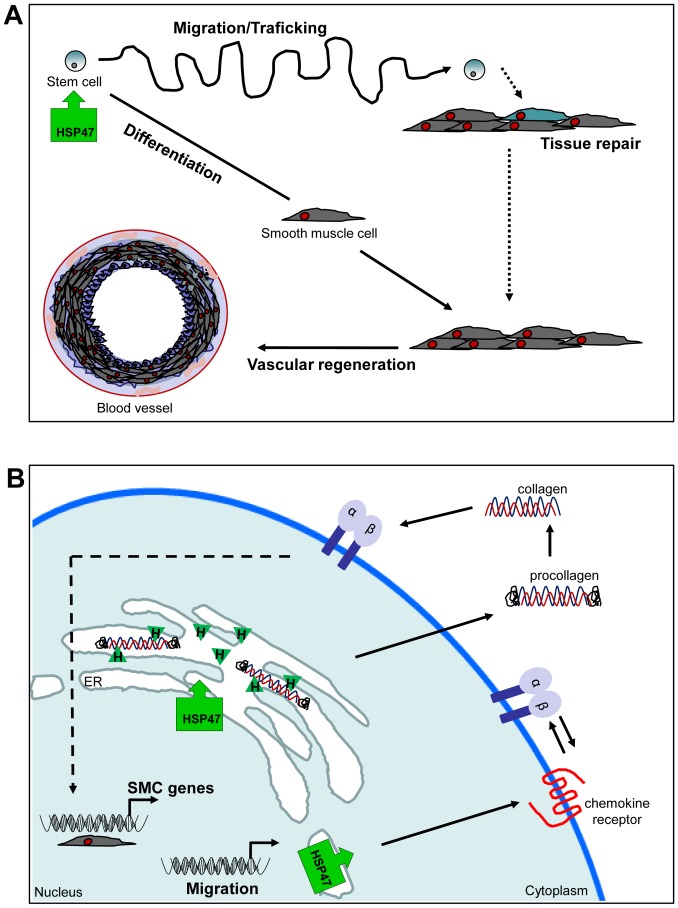
Schematic illustration of the role of HSP47 in enhancing ESC-SMC differentiation and chemotaxis. The over-expression of HSP47 results in an increase of both ESC chemotaxis and SMC differentiation that can lead to tissue repair and vascular regeneration (A). The over-expression of the ER-resident HSP47 (green block arrow) induces SMC differentiation by preferentially binding to procollagen molecules, assists in their functional maturation and subsequent release into the extracellular space of ESC. Increased secretion of collagen can in turn induce SMC differentiation by the activation of SMC specific genes via integrin stimulated signal pathways. The over-expression of HSP47 (green block arrow) also indirectly induces a panel of chemokine receptors, potentially through the co-operation with integrin signaling pathways (B).

While evaluating stem cell chemotaxis in response to various chemokines, we also considered the potential effects of HSP47 over-expression on stem cell chemokine receptor expression. Using ESC from 3 separate preparations (in the absence of any stimulus or genetic manipulation), we observed that the cells expressed variable gene levels of chemokine receptors; CCR1-5, CCR7, CXCR1 and CX3CR1 were detectable (Figure 4C). Following the over-expression of HSP47, we found a marked increase in the gene expression levels of most, if not all, chemokine receptors (Figure 4D). Interestingly, chemokine receptors CCR3, 4, 6 and 9, CXCR4 and CX3CR1 were significantly upregulated by at least 3 folds as compared to the mock controls (Figure 4D). Together, our study provides first evidence that HSP47 can play a putative role in driving stem cell-SMC differentiation and enhancing their chemotactic capacities through the induction of chemokine receptors (Figure 5A). These data indicate a potential avenue in which stem cell therapy for vascular diseases could be exploited and improved for clinical use.

## Discussion

Vascular SMC are critical for the maintenance of homeostasis within normal blood vessels and are often compromised as a result of insult from vascular diseases. In the recent decades, many investigators have strived to identify numerous therapeutic strategies to engineer vascular tissues that can efficiently replace the lost cells [2]. While the exploitation of ESC as a cell source for functional SMC is extremely attractive [6], [7], [22], [25], [26], there remains the need for improvement of their efficacy in clinic. In our study, we provide the first evidence that HSP47 gene expression is significantly upregulated during the differentiation of ESC into SMC. Although predominantly known as an ER-expressed protein, HSP47 has also been reported to be expressed on the cell surface of several epidermoid carcinoma lines [27]. Interestingly, HSP47 was also detected in the peripheral blood of patients with rheumatic autoimmune disease [28], suggesting that it is also expressed as a secreted protein. In our study, we found a distinct pattern of HSP47 expression that was restricted within the ER of differentiating ESCs, and not on the cell surface. Furthermore, our study confirms that the lack of functional HSP47 results in an attenuation of ESC-SMC differentiation altogether.

Subsequent experiments demonstrated that the over-expression of HSP47 alone (i.e. in the absence of exogenous stimulants) was sufficient to induce a marked increase in ESC-SMC differentiation. Although the precise mechanisms that drive ESC-SMC differentiation via HSP47 over-expression remain to be elucidated in forthcoming studies, it is likely that it is facilitated by an increase in the production and secretion of procollagens [29]. Indeed, collagen (types I and IV) have been shown to induce SMC differentiation of ESC via integrin (α1/β1/αV) signaling [22]. Furthermore, Matsuoka et al. demonstrated that ESC derived from HSP47-null mice resulted in dysfunctional type I and type IV collagens that lead to abnormal structural formation of embryoid bodies [30].

Transdifferentiation of stem cells is often coupled with efficient trafficking and directed migration of the cells towards a specific target tissue to ensure that a successful initiation of tissue repair and regeneration can be carried out. Moreover, the capacity of stem cell chemotaxis is also crucial for functional integration during which the cells are injected directly into a site of injury. Chemokine-mediated signalling is fundamental in the migratory behaviour of many cell types and is upregulated especially in response to tissue injury or pathological conditions [9], [10]. In the absence of exogenous manipulation, we showed that ESC can migrate towards the bottom of 8.0 µm transwells in response to a restricted set of chemokines such as eotaxin, KC and SDF-1. It is noteworthy that, except for SDF-1, the migration towards the chemokines was either relatively weak or undetectable when compared to controls. The weak affinity of their migratory response was not surprising owing to the variable and low expression of chemokine receptors that the ESCs express, namely CCR1-5, CCR7, CXCR1 and CX3CR1. These data provide potential evidence as to why translational and clinical studies using stem cells as therapeutic agents remain variable and controversial [31], [32].

To date, our data comprise the first indication that the over-expression of HSP47 results in a significant induction of ESC chemotaxis. More importantly, the over-expression further induced ESC response towards the panel of chemokines and this was concomitant with an upregulation of most chemokine receptors, except for CCR1 and CCR2, by at least 2 folds compared to the controls. Indeed, the over-expression of chemokine receptors such as CXCR4 [33] and CCR1[34] in mesenchymal stem cells (MSC) was shown to result in increased cellular migration and survival. Mechanistically, the induction of ESC chemokine receptor expression is unlikely to be a direct result of HSP47 over-expression because HSP47 is known to bind solely to collagens of at least types I-V in the ER [14], [35]. It is plausible that the increase in ESC chemotaxis is mediated by a co-operation between the chemokine receptor and integrin signaling pathways [36], [37] (Figure 5B). The precise mechanisms involved remain to be elucidated.

In summary, the present study provides fundamental evidence that HSP47 is a ER-resident protein that is highly expressed and required during SMC differentiation of ESC. Furthermore, our data provides evidence that the expression of HSP47 can be manipulated in ESC to induce their active chemotaxis and differentiation towards the SMC lineage in the absence of exogenous stimulation. Our simple but concise study postulates a novel and promising approach to improve stem cell therapy for vascular repair and regeneration.
